# Performance predication of a solar assisted desiccant air conditioning system using radial basis function neural network: An integrated machine learning approach

**DOI:** 10.1016/j.heliyon.2024.e29777

**Published:** 2024-04-17

**Authors:** Sibghat Ullah, Muzaffar Ali, Muhammad Fahad Sheikh, Ghulam Qadar Chaudhary, Laoucine Kerbache

**Affiliations:** a Mechanical Engineering Department, University of Engineering and Technology, Taxila, Pakistan; b Department of Mechanical Engineering, University of Management and Technology, Sialkot Campus, Lahore, Pakistan; c Division of Engineering Management and Decision Sciences, College of Science and Engineering, Hamad Bin Khalifa University, Doha, Qatar; d Mechanical Engineering Department, Mirpur University of Science and Technology, AJK, Pakistan; e Division of Engineering Management and Decision Sciences, College of Science and Engineering, Hamad Bin Khalifa University and HEC Paris, Doha, Qatar; f Department of Thermal Science and Energy Engineering, University of Science and Technology China, China

**Keywords:** M-cycle, Desiccant, Evaporative cooling, Solar thermal system, Artificial neural network

## Abstract

In this Paper solar desiccant air conditioning system integrated with cross flow Maisotsenko cycle (M-cycle) indirect evaporative cooler is used to investigate the performance of whole system in different range of parameters. Solar evacuated tube electric heater is used to supply the regeneration temperature to the desiccant wheel, whereas, Desiccant Wheel (DW) and M-cycle is used to handle latent load and sensible load separately. Major contribution of this research is to predict system level performance parameters of a Solar Assisted Desiccant Air Conditioning (Sol-DAC) system using Radial Basis Function Neural Network (RBF-NN) under real transient experimental inlet conditions. Nine parameters are mainly considered as input parameters to train the RBF-NN model, which are, supply Air temperature at the process side of desiccant wheel, supply air humidity ratio at process side of the desiccant wheel, outlet temperature from the desiccant wheel at process side, outlet humidity ratio from the desiccant wheel at process side, regeneration temperature at regeneration side of the DW, outlet temperature from the heat recovery wheel at process side, outlet humidity ratio out from the Heat Recovery Wheel (HRW) at process side, temperature before heat recovery wheel regeneration side of the system, humidity ratio before heat recovery wheel regeneration side of the system. Four parameters are considered as the output of the RBF-NN model, namely: output temperature, output humidity, Cooling Capacity (CC), and Coefficient of Performance (COP). The results of the RBF-NN model shows that the best Mean Squared Error (MSE) and Regression coefficient (R) for outlet temperature prediction are 0.00998279 and 0.99832 when regeneration temperature is 70 °C and inlet humidity at 18 g/kg. Best MSE and R for predication of outlet humidity are 0.0102932 and 0.99485 when the regeneration temperature is 70 °C and inlet humidity at 16 g/kg. Best MSE and R for predication of COP are 0.0106691 and 0.9981 when the regeneration temperature is 70 °C and inlet humidity 12 g/kg. Best MSE and R for predication of CC are 0.0144943 and 0.99711 when the regeneration temperature is 70 °C and inlet humidity 14 g/kg. Experimental and predicted performance parameters were in close agreement and showed minimal deviation. Investigations of predicted results revealed that trained RBF-NN model was capable of predicting the trend of output result under the varying input condition.

## 1 Introduction

Last few years have witnessed a huge increase in energy demands for air conditioning loads due to improvements in life quality, rapid urbanization and to fulfill the requirements of human thermal comfort levels. Most of the air conditioning demands are being fulfilled through conventional air conditioning systems like vapor compression-based air conditioning systems. In vapor compression systems air is cooled below the dew point and condense the moisture present in the air. To achieve desire humidity level this cooled air is dehumidified, and in next process reheated to the desired indoor temperature which cause the higher energy consumption. These conventional air conditioning systems have certain drawbacks including high electricity consumption and uses refrigerants like Chlorofluorocarbons (CFCs) and Hydrochlorofluorocarbons (HFCs) which cause global warming and ozone layer depletion [1]. Due to their greenhouse emissions, there is a dire need for sustainable, eco-friendly, and low-cost alternate air conditioning solutions.

Desiccant Air Conditioning (DAC) systems have gained immense interest as a substitute for traditional air conditioning systems due to their efficient use of thermal energy [2,3]. DAC systems allow higher ventilation air flow to enhance indoor air quality [4], ensuring reduced humidity level and eliminating airborne contaminants [5]. In Solid DAC systems, mainly rotary desiccant wheels are employed for dehumidification purpose and have great potential to control latent load. Latent load is handled by the desiccant wheel, and sensible load is controlled through Direct Evaporative Cooler (DEC) or Indirect Evaporative Cooler (IDEC) according to the kind of working conditions, such as dry or humid [6,7]. Integration of DEC with the desiccant system has a problem adding moisture in dry air which comes from the desiccant wheel. This type of system may fail in rainy sessions or climates where the humidity level is over 18 g/kg. To overcome this problem, M-cycle (IDEC) can be a good option as no water content is added to the process air, making them a more attractive option than DEC systems in humid climates [[8], [9], [10]].

The heat and mass transfer between two air streams is the base of the M-cycle's working theory. There are two air streams in the M-cycle: one is a dry air stream (product), and the second is a wet air stream (working stream). In the M-cycle, the energy is absorbed in the form of latent heat and released during water evaporation from the wet stream of air. The inlet air is divided into two paths, one is dry channels that are used for the supply of cooled product air. Whereas second path is termed as wet channels and in these channels product's energy will evaporate and be dissipated as the working stream travels through the moist path [9].

Ma et al. [10] studied the effectiveness of different cooling systems powered by solar energy at various cities of Australia and reported that solar driven DAC offers promising economic benefits specially in hot and humid regions such as Brisbane and Darwin. Kousar et al. [11] estimated the performance of DAC system combined with two DEC coolers on the supply and return side. Performance evaluation revealed the maximum Coefficient of Performance (COP) to be at 0.49 and Energy Efficiency Ratio (EER) value of 3. Fong et al. [12] presented the effectiveness of an absorption system combined with Desiccant Wheel (DW) use in warm and tropical region of Hong Kong. Solar thermal energy was employed to drive absorption and DEC cycle. The results showed that the proposed design can reduce 50 % of primary energy usage when compared to vapor compression systems. Al-Alili et al. [13] performed experimentation on desiccant cooling system integrated with vapor compression system, resulted in better COP when compared with freestanding vapor compression system. Zhan et al. [14] studied the performance of two different types of M-cycle arrangement which are cross flow and counter flow. Results revealed that the counter flow arrangement had 20 % more Cooling Capacity (CC) compared with cross flow arrangement under similar conditions. Pandelidis et al. [15] conducted numerical study on DW coupled with cross flow heat exchanger for moderate climatic conditions of central Europe. Numerical study indicates that COP of this proposed system is 60–65 % higher than the traditional one. Chaudhary et al. [16] studied the performance of a solar powered DAC system integrated with IDEC and concluded that stand alone IDEC was not able to fulfil the human comfort level integration of desiccant system enhance the system performance and helped to control the latent and sensible load. Jani et al. [17] studied on the TRNSYS software for the simulation of the solid desiccant system assisted with solar energy. TRNSYS software have potential to optimize the desiccant system performance due to transient behavior in nature and in thermal system it has good agreement within acceptable error bands.

Recently, the artificial neural network (ANN) has predicted the performance of thermal energy systems more accurately than conventional regression methods. For instance, Jani et al. [18] used the ANN model to predicted the performance of desiccant dehumidifier. Thermal system such desiccant wheel needs complex mathematical model to predict the performance, instead to solve the complex numerical equation it is best to use ANN models to resolve the solutions. To predict the desiccant system performance dry bulb temperature, humidity ratio, regeneration temperature and air flow rate considered as input features and outlet humidity ratio, temperature and moisture removal capacity taken as output features of the system. Performance predication of the system showed close agreement between experimental results and predication results. The temperature and specific humidity of air supplied at outside from the DW was predicted by ANN using five features. Correlation coefficient (R) was found to be 0.986 for both outlet parameters that indicates good close correlation between the estimated data of the model and experimental data [19]. Similarly, in another study, a multivariant regression analysis approach was adopted to develop ANN model to optimize the marine desiccant air conditioning system. Results revealed that the regression coefficient (R^2^) of model achieved 0.999 and the estimated error being substantially less than 1 % [20].

Tariq et al. [21] used deep learning approach of Bayesian Regularization algorithm to predict the total water footprint (TWF) and CC of the desiccant system integrated with DEC by considering five input features ambient temperature, ambient relative humidity, regeneration temperature, supply flow rate and regeneration flow rate of the air. Prediction results showed the value of the coefficient of determination are 0.98856 and 0.99246 for CC and TWF, respectively. Güzelel et al. [22] proposed the decision tree (DT) method for desiccant wheel by considering six input parameters and compared results with multilayer perceptron regression and multiple linear regression, DT method showed best results among of them. Cerci et al. [23] used multiple linear regression techniques and activation function of ANN model on desiccant system and by comparing results with each other it has been observe that MLR results are best among them. Motaghian et al. [24] considered the length of channel, rotational speed, purge angle and area ratio of process/regeneration for desiccant wheel are all comprehensive design characteristics that are considered as inputs of the ANN model. Comino et al. [25] studied the multi-task ANN model for the predication of humidity and temperature for standalone DW and presented the result of comparative study. Moisture removal rate and total efficiency considered as performance indicators of DAC system by collecting the experimental data results are used to train the ANN model. A close agreement is observed between the experimental results and performance prediction through ANN model.

Extensive literature review indicates that most of the researchers focused on simulation and numerical based studies to evaluate the solar desiccant air conditioning systems. One of the main reasons is such a system testing need high initial cost and need high skill to operate these systems. Moreover, mostly desiccant systems are integrated with DECs however limited practical studies have focused on integration of solid desiccant system with M-cycle (IDEC). Furthermore, using ANN approach to predicate the system performance at system level is very rare in the published literature. Mostly worked is carried out in recent past has been focused on the prediction of performance parameters of DAC systems on component level. In order to improve the performance of DAC systems, limited studies have focused on the integration of solar DAC system with M-cycle (IDEC) and subsequently the literature lacks the performance parameter prediction of a Sol-DAC system at a system level. Energy systems such as Sol-DAC are mainly designed to comply with human thermal comfort levels which makes the performance parameters of the whole experimental data concentrated around a focus point. Predicted performance parameters in such of energy systems should be in close agreement with the experimental values, that requires the need of a regressor with high predication performance. Based on the shortcomings of the above literature, a machine learning based data driven model has been established to predict the performance parameters of the designed Sol-DAC system. Table 1 presents the comparison of recent work done by different authors.

**Table 1:** Summary of the literature studies.

Author	Year	Nature of work		Machine Learning Techniques used	Regression	MSE
		Experimental	Simulation			
Present	2023	**✓**		Radial Basis Function Neural Network (RBF-NN)	R_T_ = 0.99832	MSE_T_ = 0.00998279
					R_ω_ = 0.99897	MSE_ω_ = 0.0102932
					R_COP_ = 0.9981	MSE_COP_ = 0.0106691
					R_CC_ = 0.99711	MSE_CC_ = 0.0144943
Tariq et al. [21]	2022	**✓**		Multi-Layer Perceptron Artificial Neural Network (MLP-ANN)	R^2^ = 0.98856	0.0057
Güzelel et al. [22]	2021		**✓**	Multiple Linear Regression	R^2^_T_ = 0.9986	RMSE_T_ = 0.3295
					R^2^_ω_ = 0.9994	RMSE_ω_ = 0.0995
Çerçi et al. [23]	2020		**✓**	Multiple Linear Regression (MLR)	R^2^_T_ = 0.99996	RMSE_T_ = 0.07562
					R^2^_ω_ = 0.99998	RMSE_ω_ = 0.02344
Motaghian et al. [24]	2020	**✓**		Artificial Neural Network (ANN)	R = 0.99	MSE = 0.000059
Comino et al. [25]	2019	**✓**		Multitask Artificial Neural Networks (ANNs)	–	MSE = 0.390
Zendehboudi et al. [26]	2016		**✓**	Genetic Algorithm Least-Squares Support Vector Machine (GA-LSSVM)	R^2^_T_ = 0.9978	RMSE_T_ = 0.2074
					R^2^_ω_ = 0.9998	RMSE_ω_ = 0.031
UÇKAN et al. [27]	2015	**✓**		Artificial Neural Network (ANN)	R_t_ = 0.9860	MSE_T_ = 0.54
					R_ω_ = 0.9865	MSE_ω_ = 0.18
Panaras et al. [28]	2010	**✓**		Effectiveness Parameters	–	RMSE_T_ = 1.24 RMSE_ω_ = 0.30

## 2 Proposed technique

**Fig. 1:**
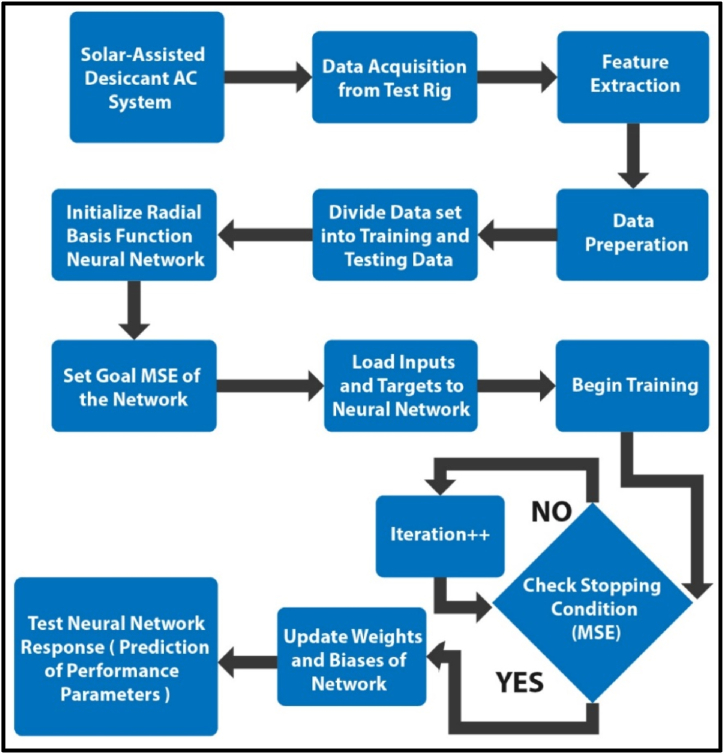
Proposed technique flow chart using RBF-NN.

Fig. 1 shows the flow diagram of the proposed supervised learning technique that uses RBF-NN as regression tool to predict the performance output parameters of Sol-DAC system. An extensive experimental study was designed and performed to test the installed system at a range of input ambient conditions. Data acquisition was performed with multiple sensors to note the reading of temperature and humidity which were located at all inlet and outlet points for each component of the installed system. By examining the data which acquired from the acquisition system, different input parameters which have potential to affect the system output performance considered as distinct features and this data fed as inputs to the RBF-NN. Dataset was divided into training and testing data. To attain a predetermined Mean Squared Error (MSE), the ANN is trained using training data for as many iterations as possible MSE. After the target MSE is achieved, network is trained, and the weights are then freeze. The performance parameters of Sol-DAC are then predicted using testing data by this trained neural network.

## 3 System description

### 3.1 Integrated solar powered desiccant air conditioner

**Fig. 2:**
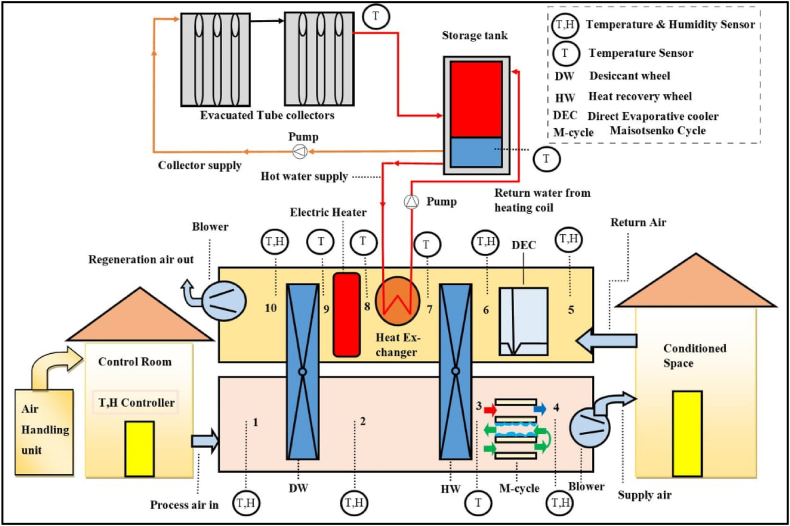
Schematic diagram of Sol-DAC.

A solar desiccant air conditioner typically consists of solar thermal collectors, storage tank, desiccant dehumidifier, heat wheel, IDEC, auxiliary electric heater and two supply and return air fans. Fig. 2 represents the schematic diagram of DAC system. Solar collectors are used for water heating that was used in heating coils to attain the required regeneration temperature. The Solar powered Desiccant Air conditioner (Sol-DAC) system consists of two distinct air streams supply air stream and return air stream. On process side humid and hot air is passed through a DW which dehumidify the air stream whereas the temperature is increased (stage 1-2). After dehumidification of air this hot and dehumidified air is further passed through the heat recovery wheel (HRW) which subsequently cool down (stage 2-3). This partially cooled and dry air is then drawn through an indigenous developed IDEC based on M-cycle which reduces the temperature up to desired thermal comfort (stage 3-4). Whereas, on the return section of test rig the exhaust air which is comes from the conditioned space/ambient air moves into the cooling system at stage 5 and then passes DEC, temperature of air drop here and exits at stage 6. DEC was installed between 5 and 6 as when air passed through the desiccant wheel at point 1 air gain dehumidified due to silica gel based desiccant wheel and got temperature high at point 2. To drop this high temperature before enter into the M-cycle (IDEC) a heat recovery wheel is installed at point 2 between the desiccant wheel and M-cycle. HRW act as rotary heat exchanger, to supply cool air into the heat recovery wheel DEC was installed at regeneration of the system. HRW acquired cool air from DEC and due to rotation of HRW, exchange the temperatures of air at point 2 and point 6. Following that from 6 to 7 there will a sensible heat exchange, among the process and return air streams. To attain the regeneration temperature air is passed through to air to water heat exchanger from 7 to 8. If required regeneration temperature is not attained from the solar collector, an auxiliary electric heater is used from 8 to 9. At last, the heated air desorbs the DW and moves out from the system at stage 10.

## 4 Experimental setup

**Fig. 3:**
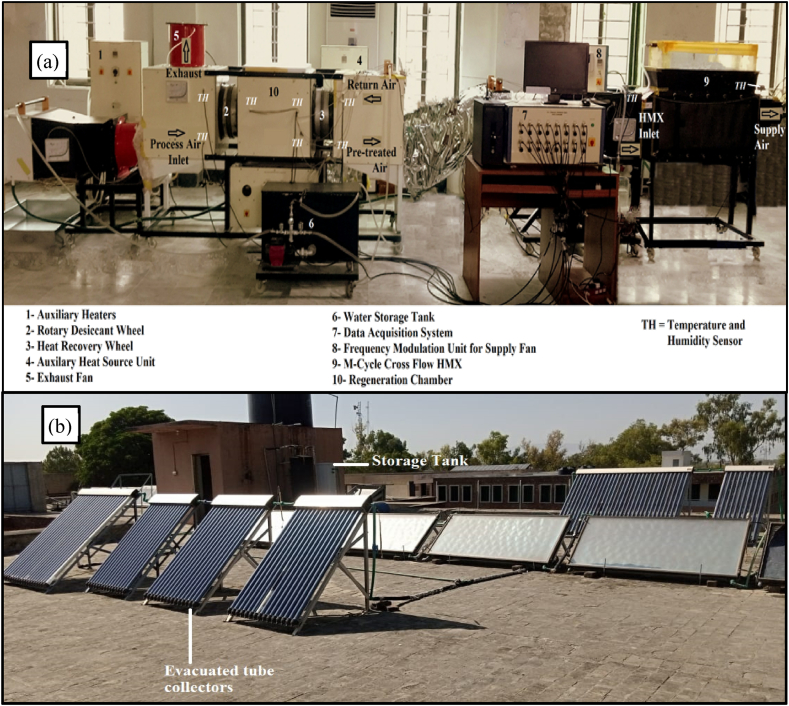
**(a)** Experimental setup of Sol-DAC integrated with M-cycle***;* (b)** Solar collectors integrated with Sol-DAC system.

The experimental arrangement of the DAC system is shown in Fig. 3(a) and (b). The experimental test rig consists of rotary DW, HRW, M-cycle Indirect cross flow HMX (IDEC), driving motors, data logger, measuring instruments, water pumps, and fans. The designed specification of equipments installed in the experimental rig is illustrated in Table 2.

**Table 2:** Design specifications of the experimental setup.

Desiccant Wheel	
Material of DW	Silica gel
Thickness of DW (mm)	200
Diameter of DW (mm)	370
Capacity of air handling	450 CFM
**Heat Recovery Wheel**	
Material	Aluminum
Thickness of HRW (mm)	200
Diameter of HRW (mm)	370
Sensible effectiveness	0.75
**M-Cycle IDEC**	
Height (m)	0.48
Length (m)	0.90
Width (m)	0.28
Thickness of wall (mm)	0.3
Type of Channel	Rectangular
Channel width (mm)	5
Channel height (mm)	25
Thickness of wick (mm)	0.1
Motor power for rotation of the wheel	0.75 kW
Fan motors power (supply air & return air)	0.75 kW
**Solar Collectors**	
Area of flat plate solar collectors	7.2 m^2^
Area of evacuated solar collectors	4.7 m^2^
Volume of water storage tank	0.3 m^3^

Sol-DAC system performance was tested using the different measurements available to record the temperature and humidity of air at different points of the test rig. K-type sensors temperature sensors and KLK 100 humidity sensors were installed. To record the water temperature at the inlet and outlet of the solar system, K-type thermocouples were installed. Solar radiations were recorded by the pyranometer. Details of instruments used to test the performance of the test rig are given in Table 3.

**Table 3:** Measuring instruments specifications.

Sr#	Measurement	Measurement instrument	Measuring range	Accuracy
1	Temperature	K-type	0–100 °C	±0.15 °C
2	Relative humidity	KLK 100	0–99 %	±2 %
3	Air flow rate	Anemometer	0.1–20 m/s	±0.01 m/s
4	Solar radiation	Pyranometer	0–1500 W/m^2^	1 W/m^2^
5	Water temperature	K-type Thermocouple	0–200 °C	0.3 %
6	Water flow rate	Flow transducers	0.1–120 LPM	0.5 Lpm

### 4.1 Experimental methodology

The whole Sol-DAC system was tested to examine the impact of different inlet ambient conditions, regeneration temperature and air flow rate. Performance analysis of Sol-DAC integrated with M-cycle was examined by changing the design conditions like humidity ratio, temperature, and regeneration temperature, which were supplied to the system. Input conditions at the supply side are humidity ratio, regeneration temperatures, temperature, air flow rates, and return air was supplied at control conditions and were used to test the system under different scenarios. Table 4 provides a list of the range of analytic parameters that were used.

**Table 4:** Parameters for testing.

Operating variables	Range
Supply air temperature at the process side (°C)	25–45
Supply Humidity ratio at process side (g/kg)	12–18
Temperature of inlet air at regeneration side (°C)	26–30
Regeneration air temperature (°C)	60–80
Humidity of inlet air at regeneration side (g/kg)	12–14
Speed of air at process side (kg/h)	650
Speed of air at regeneration side (kg/h)	650

A pre-conditioner was used to create the atmospheric conditions for the supply side of system conditions (humidity and temperature) at the intake of DW, and the return air conditions are maintained at the inlet of HRW. This pre-conditioner had electric heaters, a humidifier, and sensors for humidity and temperature. The experimental arrangement of Sol-DAC was operated under the different inlet parameters and waited till different inlet conditions that were supplied to the system were stable and established properly. Under a specific parametric range, the data collection system was operated until the scenario of the design experiment was properly established. DW installed in the test rig controls the latent load of the process air by absorbing the moisture content present in the process air, and M-Cycle (HMX) installed to control the sensible load of the process air without adding moisture in the process air; both of these components installed control the latent and sensible load separately. The experimental equipment was returned to its initial settings following the completion of each scenario, and the same procedure was carried out for the subsequent scenario. From medium to high humidity ratio was examined in this investigation. According to the input ambient conditions, a regeneration temperature range of 60–80 °C was used for the whole analysis. The solar system contributed 70 % of the total regeneration temperature to regenerate the desiccant wheel. It is crucial to note that this study focuses on evaluating the integrated MC-DAC system's overall performance compared to a traditional one, not on the creation of regeneration heat. Table 5 provides a comparison between the present study and studies found in the literature.

**Table 5:** Predicted output features on modeling a DAC system using ML techniques.

Authors	Year	Temperature	Humidity	COP	CC
Present	2023	**✓**	**✓**	**✓**	**✓**
Tariq et al. [21]	2022	✕	✕	✕	**✓**
Güzelel et al. [22]	2021	**✓**	**✓**	✕	✕
Çerçi et al. [23]	2020	**✓**	**✓**	✕	✕
Motaghian et al. [24]	2020	**✓**	**✓**	✕	✕
Comino et al. [25]	2019	**✓**	**✓**	✕	✕
Zendehboudi [26]	2016	**✓**	**✓**	✕	✕
UÇKAN et al. [27]	2015	**✓**	**✓**	✕	✕
Panaras et al. [28]	2010	**✓**	**✓**	✕	✕

## 5 Artificial neural network

An information processing system that resembles a biological brain is called an ANN. The computational model is composed of a number of interconnected neurons. Depending on supervised learning and unsupervised learning, neural network architecture can be determined. The network is fed input patterns for supervised learning along with the associated target signal. The error signal indicates the change between the output and the target. The error is then continuously adjusted by the weights against the target signal until it is at a certain level. Without a target signal, neural networks classify signals into distinct groups based on shared unsupervised learning; ANN classifies signals into two separate classes by considering features that are similar without needing a target signal. The training phase of the network is the period during which weights are adjusted. The recall phase of the network is testing the network against new input patterns after training. The structure of RBF-NN is the same as multilayer feed forward networks, which have three-layer neural networks: input layer, hidden layer, and output layer. There is a hidden layer between the input layer and the output layer, and the input layer has ‘n' neurons while the output layer has ‘m' neurons. RBF-NN is a simple tool for classification, and it may also be used to approximate functions using Gaussian potential functions. Using the training algorithm listed below, RBF-NN is trained. Utilizing the Gaussian activation function, the output is calculated. Such a function provides a non-negative answer for all values of y [29].

### 5.1 Radial basis function neural network

The radial basis approach is a multilayer feed-forward neural network having input, hidden, and output layers. The main difference between back propagation and radial basis function neural networks is that radial basis uses the Gaussian potential function as its activation function. The following equations' functions are recognized as radial basis functions.

(1) δ(r)=r

(2) $$\delta \left(r\right)=r^{2}$$

(3) $$\delta \left(r\right)=r^{3}$$

(4) $$\delta \left(r\right)=\exp\left(-r^{2}\right)$$

**Fig. 4:**
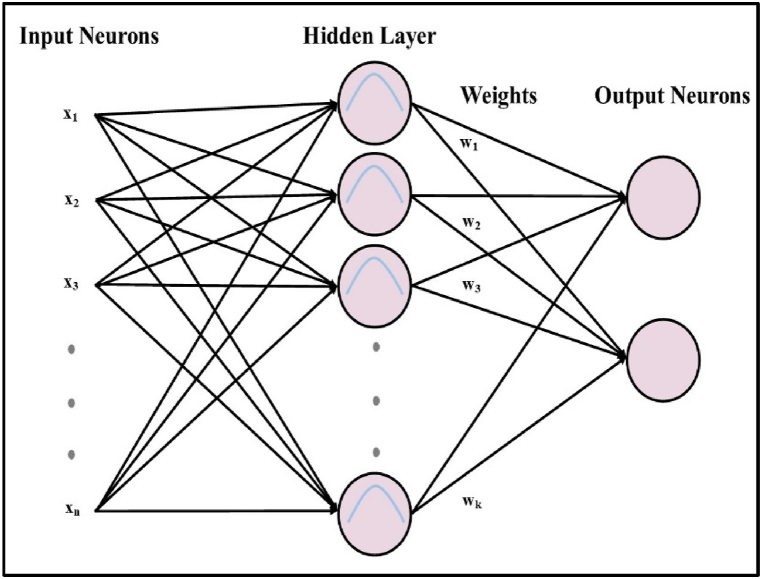
Radial basis function neural network with n hidden neurons.

The number of neurons in the input layer is “m,” the number of neurons in the output layer is “n,” and a hidden layer is sandwiched between the input layer and the output layer. These numbers correspond to the characteristics of the dataset and the number of output classes, respectively. The first two layers, the input layer, and the hidden layer have hypothetical connections, but the hidden layer and the output layer have weighted connections. Fig. 4 depicts the architecture of a radial basis function neural network.

The use of radial basis function neural networks makes classification simple; in addition, these networks can be used to approximate functions using Gaussian potential functions [30]. Below is the radial basis function neural network training algorithm [31]. The radial basis function utilizes the Gaussian activation function. Such a function provides a non-negative response for all values of r. The same function as the equation is used.

(5) $$G\left(x\right)=\exp\;\left(-x^{2}\;\right)$$

The derivative of the function is calculated as

(6) $$G^{\prime }\left(x\right)=-2xexp\left(-x^{2}\right)$$

(7) $$G^{\prime }\left(x\right)=-2xG\left(x\right)$$

After defining the RBF-NN architecture, small random values are assigned to weights. Each input unit $\left(y_{i},i=1,\ldots ..,n\right)$ receives input signals after which the Radial basis function is calculated for each input. The set of input vectors is utilized for choosing the Radial basis function centers. For a guaranteed adequate sampling of the input vector space, sufficient numbers of centers must be designated. The output of i_m_ unit G_i_(x_i_) in the hidden layer

(8) $$G_{i}\left(x_{i}\right)=\exp\left(-\sum_{j=1}^{r}{\left[x_{\text{ji}}-x_{\text{ji}}^{\wedge }\right]}^{2}/\mu_{i}\right)$$

whereas $x_{ji\;}=Centre\;of\;the\;RBF\;unit\;for\;input\;variables,\mu_{i}=Width\;of\;the\;ith\;RBF\;unit,x_{ji\;}=j_{th}\;variable\;of\;input\;pattern\text{.}$ Output of the RBFNN model is calculated as follows

(9) $$H_{net}=\sum_{i=1}^{N}w_{im\;}G_{i}\;\left(x_{i}\right)+Z_{o}$$

whereas, N = number of hidden layer nodes, $H_{net}$ = Output value of mth node in output layer for the nth incoming pattern. $w_{im\;}$ = Weight between m_th_ output node and i_th_ radial basis function unit. $Z_{o}$ = Biasing term at nth output node. Changing of weights and number of iterations can be the stopping criteria for training of the network.

## 6 Results and discussion

The analysis of experimental data showed that a sample of 720 data points was the most suitable for training the RBF-NN model. The RBF-NN model was able to identify the weights and bias values that reduce the difference between the output of the neural network and the experimental values of the system. During the training phase, the training data was positioned at the input layer. The output neurons layer and a single hidden layer were used to predict the output features. The performance of the network was significantly impacted by the weights that were assigned to each neuron.

The network's input neuron count is the same as the total number of data features. The output classes define the number of output neurons. To optimize the network performance, the number of hidden layer neurons can be varied. The output layer is completely interconnected with the hidden layer, which is also coupled to the input layer. All of these connections have weights that amplify the input, and the neurons in the hidden layer are capable of activation. Experimental data acquired from the installed Sol-DAC system for a range of input ambient conditions was processed to select the distinct features. Separate RBF-NN models were developed for each performance parameter, which are output temperature (T_out_), output humidity (ω_out_), CC (kW), and COP of the Sol-DAC. RBF-NN was employed to predict the output features of a Sol-DAC due to its high generalization, approximation, and regression capabilities. The main difference between radial basis function neural networks and other feed-forward neural networks is that radial basis uses Gaussian potential function as its activation function in hidden layer neurons. The architecture of the developed RBF-NN model for each performance parameter depends upon the fed input features for that specific case. Hidden layer neurons of the RBF-NN use Gaussian functions as its activation function. The dataset was divided into training and testing datasets. 70 % of the dataset is used for training the network, and 30 % of the dataset is used against validation and testing of the developed network. From this, 30 % of the testing dataset, 50 % samples were utilized for testing of the network response and 50 % samples were utilized for validation. RBF-NN was trained against the preset number of neurons and a preset goal MSE as a stopping criterion of the training data. For all the individual RFB-NN models, Goal MSE was selected as 0.01, and the maximum neurons were selected as 1000 to avoid excessive computational difficulties and delays. The spread is a crucial hyperparameter that controls the sensitivity of RBF neurons in shaping their response and has a significant effect on the ability to capture data features and the generalization capability of the trained network. Selecting a significant value of spread can make the model overly sensitive to noise and outliers, and a relatively small value of spread can limit the ability to generalize beyond training data. Balancing spread is essential for generalization to unseen data; therefore, its value is selected a 1.

The network is trained until it is able to meet the stopping criteria; optimized weights at this stage are then fixed. This trained neural network is then used to predict the performance parameters of a Sol-DAC system with testing data. RBF-NN is tested against testing and validation data using updated weights after the network has been trained. The designed RBF-NN model architecture was constructed and trained using MATLAB software. The details of the training parameters used in the present study are listed in Table 6.

**Table 6:** Training parameters for RBF-NN.

Parameter	Value
Goal MSE	0.01
Maximum Neurons	1000
DF	25
Spread	1

To evaluate the generalization capabilities of the feed forward model of RBF-NN based on the criteria of Mean Square Error (MSE) and Correlation Coefficient (R). Equations (10), (11) presented the mean square error and correlation coefficient (R), respectively [26].

(10) $$MSE=\frac{1}{n}\sum_{i=1}^{N}{\left(Y\begin{array}{c} act\\ i \end{array}-Y\begin{array}{c} pre\\ i \end{array}\right)}^{2}$$

where in equation of mean square error MSE n, $Y\begin{array}{l} act\\ i \end{array}$, $Y\begin{array}{l} pre\\ i \end{array}$, and $Y^{\bar{act}}$ presented the total number of data, ith actual data, ith predicted data via the model and average of actual data, respectively [24].

(11) $$R=\frac{\sum_{i=1}^{N}\left(I_{actual,i}-{\bar{I}}_{actual})(I_{predicted,i}-{\bar{I}}_{predicted}\right)}{\sqrt{\sum_{i=1}^{N}{\left(I_{actual,i}-{\bar{I}}_{actual}\right)}^{2}\sum_{i=1}^{N}{\left(I_{predicted,i}-{\bar{I}}_{predicted}\right)}^{2}}}$$

In equation of correlation coefficient (R) $I_{actual}$ and $I_{predicted}$ denote the number of ANN outputs, the predicted data by the network, and the actual outputs, respectively whereas ${\bar{I}}_{actual}$ and ${\bar{I}}_{predicted}$ are respectively the mean values of $I_{actual}$ and $I_{predicted}$.

### 6.1 Prediction of outlet temperature (T_out_) of Sol-DAC using RBF-NN

RBF-NN was trained with a training dataset to predict the M-cycle (HMX) outlet temperature of the Sol-DAC system. To predict the M-cycle (HMX) outlet temperature, the following distinct features were fed to the RBF-NN as inputs:

• (T_1_) Supply Air temperature at the process side of DW
• (ω_1_) Supply Air humidity ratio at the process side of DW
• (T_2_) Outlet temperature from the DW at the process side
• (ω_2_) Outlet humidity ratio from the DW at the process side
• (T_9_) Regeneration temperature at the regeneration side of the DW
• (T_3_) Outlet temperature out from the HRW at process side
• (ω_3_) Outlet humidity ratio out from the HRW at process side
• (T_6_) Temperature before HRW regeneration side of the system
• (ω_6_) Humidity ratio before HRW regeneration side of the system

Table 7 presents the results of RBF-NN model trained with input distinct features of Sol-DAC system that has range of input conditions to predict the outlet temperature of the system.

**Table 7:** Summary of results of RBF-NN to predict M-cycle outlet temperature (Sol-DAC).

Humidity (ω g/kg) in	Inlet temperature (T°C)	Regeneration temperature (T°C) in	Input neurons	Output neurons	Hidden layer neuron	Goal	MSE	R
12	25–45	70	9	1	525	0.01	0.00998270	0.99049
14	25–45	50	9	1	525	0.01	0.00998279	0.963
14	25–45	60	9	1	525	0.01	0.00998279	0.77232
14	25–45	70	9	1	525	0.01	0.00998279	0.99638
14	25–45	80	9	1	525	0.01	0.00998279	0.99047
16	25–45	70	9	1	525	0.01	0.00998279	0.99071
18	25–45	70	9	1	525	0.01	0.00998279	0.99832

**Fig. 5:**
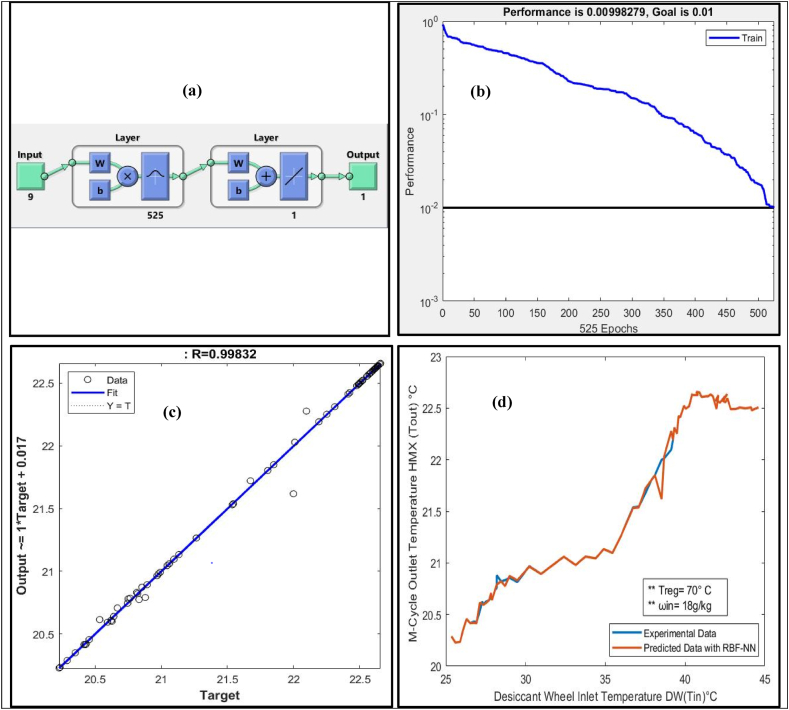
(a) Architecture of RBF-NN (b) Performance Curve (c) Regression plot (d) Comparison between Predicated and Experimental data.

RBF-NN showed the best prediction of the T_out_ measured as regression (R = 0.99832) when the supply air humidity ratio was (18 g/kg), and the regeneration temperature for the Sol-DAC system was at 70 °C. Fig. 5(a) shows the architecture of the trained RBF-NN model that consists of 9 input neurons, 1 output neuron, and chooses 525 hidden layer neurons to achieve the preset Goal MSE of 0.01. Fig. 5(b) shows the convergence curve of the RBF-NN against the preset MSE. The blue line shows the trend of reduction in MSE during training of the RBF-NN as epochs pass. It can be observed from the figure that the network took 525 epochs to achieve the preset MSE of 0.01. Fig. 5(c) shows the regression plot between the predicted output (Tout) of RBF-NN after testing and their corresponding targets from the experimental data. It is evident from the regression plot (R = 0.99832) that most of the predicted data points lies very close to the fitting line except few outliers. After predicting the T_out_ of the Sol-DAC system using trained RBF-NN, experimental and predicted data were simultaneously plotted to check the performance of the network as a regression model in Fig. 5(d). The predicted outlet temperature using RBF-NN is in close agreement with the outlet temperature of the experimental data and follows the same trend. Fig. 5(d) shows that Sol-DAC system tested in the temperature range of 25–45 °C and after air passes through the process side of desiccant wheel, heat recovery wheel and M-cycle it reduced the temperature and remain in human comfort range which is consider as 25 °C. Regeneration temperature was 70 °C for specific case to dehumidify the DW.

### 6.2 Prediction of outlet humidity (ω_out_) of Sol-DAC using RBF-NN

RBF-NN was trained with a training dataset to predict the M-cycle (HMX) outlet humidity ratio (ω_ou**t**_) of the Sol-DAC system. To predict the M-cycle (HMX) ω_out_ following distinct features were fed to the RBF-NN as inputs:

• (T_1_) Supply Air temperature at process side of DW
• (ω_1_) Supply Air humidity ratio at process side of DW
• (T_2_) Outlet temperature from the DW at process side
• (ω_2_) Outlet humidity ratio from the DW at the process side
• (T_9_) Regeneration temperature at the regeneration side of the DW
• (T_3_) Outlet temperature out from the HRW at process side
• (ω_3_) Outlet humidity ratio out from the HRW at process side
• (T_6_) Temperature before HRW regeneration side of the system
• (ω_6_) Humidity ratio before HRW regeneration side of the system

Table 8 presents the results of RBF-NN model trained with input distinct features of Sol-DAC system that has range of input conditions to predict the M-Cycle outlet humidity (ω_out_) of the system.

**Table 8:** Summary of results of RBF-NN to predict M-cycle outlet humidity (g/kg).

Humidity (ω g/kg) in	Regeneration temperature (T°C) in	Input neurons	Output neuron	Hidden layer neuron	Goal	MSE	R
12	70	9	1	601	0.01	0.0102932	0.98448
14	50	9	1	601	0.01	0.0102932	0.96469
14	60	9	1	601	0.01	0.0102932	0.84424
14	70	9	1	601	0.01	0.0102932	0.998
14	80	9	1	601	0.01	0.0102932	0.99897
16	70	9	1	601	0.01	0.0102932	0.99485
18	70	9	1	601	0.01	0.0102932	0.98647

**Fig. 6:**
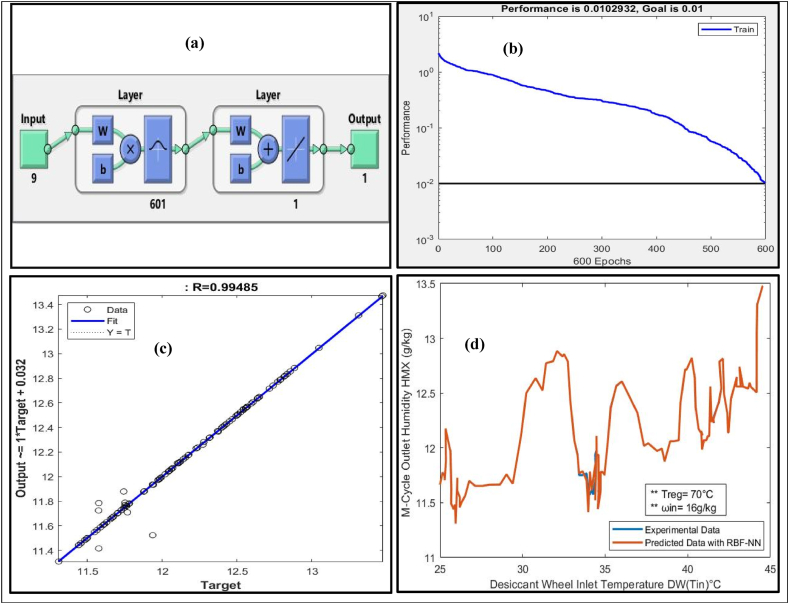
(a) Architecture of RBF-NN (b) Performance Curve (c) Regression plot (d) Comparison between predicted and experimental data.

RBF-NN showed the best prediction of the M-Cycle ω_out_ measured as regression (R = 0.99485) when the supply air humidity ratio was (16 g/kg), and the regeneration temperature for the Sol-DAC system was at 70 °C. Fig. 6(a) shows the architecture of the trained RBF-NN model that consists of 9 input neurons, one output neuron, and chooses 601 hidden layer neurons to achieve the preset Goal MSE of 0.01. Fig. 6(b) shows the convergence curve of the RBF-NN against the preset MSE. The blue line shows the trend of reduction in MSE during training of the RBF-NN as epochs pass. It can be observed from the figure that the network took 600 epochs to achieve the preset MSE of 0.01. Fig. 6(c) shows the regression plot between predicted M-Cycle ω_out_ of RBF-NN after testing and their corresponding targets from the experimental data. It is evident from the regression plot (R = 0.99485) that most of the predicted data points lies very close to the fitting line except few outliers.

After predicting the M-Cycle ω_out_ of the Sol-DAC system using trained RBF-NN, experimental and predicted data were simultaneously plotted to check the performance of the network as a regression model in Fig. 6(d). Predicted M-Cycle ω_out_ using RBF-NN is in close agreement with the M-Cycle ω_out_ of the experimental data and follows the same trend. Fig. 6(d) shows that the Sol-DAC system tested in the temperature range of 25–45 °C and humidity ratio12-18 g/kg at the inlet of the desiccant wheel of Sol-DAC. The desiccant wheel effectively dehumidified the air, reduced the humidity ratio of inlet air at the process side, and maintained the humidity ratio at 13.5 g/kg. Regeneration temperature-maintained 70 °C degree for this specific case. Regeneration temperature is a very important parameter to maintain the humidity ratio at the outlet from the system. Higher the regeneration temperature needed to dehumidify the desiccant wheel when humidity ratio at inlet of the desiccant wheel become high. Increasing the system inlet humidity required a higher regeneration temperature to provide efficient dehumidification through the desiccant wheel.

### 6.3 Prediction of COP for Sol-DAC using RBF-NN

RBF-NN was trained with a training dataset to predict the COP of the Sol-DAC system. To predict the COP, the following distinct features were fed to the RBF-NN as inputs:

• (T_1_) Supply Air temperature at process side of DW
• (ω_1_) Supply Air humidity ratio at process side of DW
• (h_1_) Enthalpy at process side of DW
• (T_2_) Outlet temperature from the DW at process side
• (ω_2_) Outlet humidity ratio from the DW at the process side
• (T_9_) Regeneration temperature at regeneration side of the DW
• (T_3_) Outlet temperature out from the HRW at process side
• (ω_3_) Outlet humidity ratio out from the HRW at process side
• (h_4_) Enthalpy at process side after the M-cycle of the Sol-DAC
• (T_6_) Temperature before HRW regeneration side of the system
• (ω_6_) Humidity ratio before HRW regeneration side of the system
• (h_10_) Enthalpy at regeneration side after the DW of the Sol-DAC

Table 9 presents the results of RBF-NN model trained with input distinct features of Sol-DAC system that has range of input conditions to predict the COP of Sol-DAC of the system.

**Table 9:** Summary of results of RBF-NN to predict COP of Sol-DAC.

Humidity (ω g/kg) in	Regeneration temperature (T°C) in	Input neurons	Output neuron	Hidden layer neuron	Goal	MSE	R
12	70	12	1	653	0.01	0.0106691	0.9981
14	50	12	1	653	0.01	0.0106691	0.96933
14	60	12	1	653	0.01	0.0106691	0.95562
14	70	12	1	653	0.01	0.0106691	0.99283
14	80	12	1	653	0.01	0.0106691	0.99451
16	70	12	1	653	0.01	0.0106691	0.98971
18	70	12	1	653	0.01	0.0106691	0.97662

**Fig. 7:**
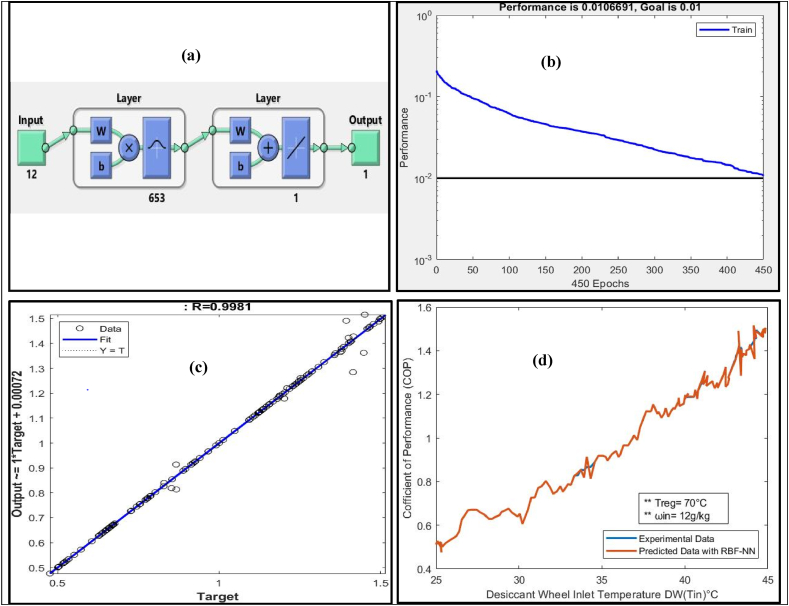
(a) Architecture of RBF-NN (b) Performance Curve (c) Regression plot (d) Comparison between Predicated and Experimental data.

RBF-NN showed the best prediction of COP of the Sol-DAC measured as regression (R = 0.9981) when the supply air humidity ratio was (12 g/kg), and the regeneration temperature for the Sol-DAC system was at 70 °C. Fig. 7(a) shows the architecture of the trained RBF-NN model that consists of 12 input neurons, 1 output neuron, and chooses 653 hidden layer neurons to achieve the preset Goal MSE of 0.01. Fig. 7(b) shows the convergence curve of the RBF-NN against the preset MSE. The blue line shows the trend of reduction in MSE during training of the RBF-NN as epochs pass. It can be observed from the figure that the network took 450 epochs to achieve the preset MSE of 0.01. Fig. 7(c) shows the regression plot between predicted COP for the Sol-DAC by using RBF-NN after testing and their corresponding targets from the experimental data. It is evident from the regression plot (R = 0.9981) that most of the predicted data points lies very close to the fitting line except few outliers.

After predicting the COP of the Sol-DAC system using trained RBF-NN, experimental and predicted data were simultaneously plotted to check the performance of the network as a regression model in Fig. 7(d). The predicted COP of the Sol-DAC using RBF-NN is in close agreement with the COP of the Sol-DAC calculated by experimental data and follows the same trend. Fig. 7(d) shows that the Sol-DAC system tested in the temperature range of 25–45 °C and humidity ratio of 12–18 g/kg at the inlet of the desiccant wheel of Sol-DAC. Regeneration temperature is a very important parameter. Higher the regeneration temperature needed to dehumidify the desiccant wheel when humidity ratio at inlet of the desiccant wheel become high. Increasing the system inlet humidity required a higher regeneration temperature to provide efficient dehumidification through the desiccant wheel. The results revealed that the COP is increased by increasing the inlet air temperature. However, there was a significant drop in the dehumidification efficiency due to the raised inlet temperature, which was supplied into the system. It is noted that the COP of the system increased when the inlet temperature of the process side is increased at constant regeneration temperature as the capacity of the system to reduce the temperature after the M-cycle is increased. The capacity to maintain the temperature at a comfort level also increases. Moreover, high inlet air temperature and high humidity have a positive effect on the COP of the Sol-DAC system.

### 6.4 Prediction of CC for Sol-DAC using RBF-NN

RBF-NN was trained with a training dataset to predict the COP of the Sol-DAC system. To predict the COP, the following distinct features were fed to the RBF-NN as inputs:

• (T_1_) Supply Air temperature at process side of DW
• (ω_1_) Supply Air humidity ratio at process side of DW
• (h_1_) Enthalpy at process side of DW
• (T_2_) Outlet temperature from the DW at process side
• (ω_2_) Outlet humidity ratio from the DW at process side
• (T_9_) Regeneration temperature at regeneration side of the DW
• (T_3_) Outlet temperature out from the HRW at process side
• (ω_3_) Outlet humidity ratio out from the HRW at process side
• (h_4_) Enthalpy at process side after the M-cycle of the Sol-DAC
• (T_6_) Temperature before HRW regeneration side of the system
• (ω_6_) Humidity ratio before HRW regeneration side of the system
• (h_10_) Enthalpy at regeneration side after the DW of the Sol-DAC

Table 10 presents the results of RBF-NN model trained with input distinct features of Sol-DAC system that has range of input conditions to predict the CC of the system.

**Table 10:** Summary of results of RBF-NN to predict CC of Sol-DAC.

Humidity (ω g/kg) in	Regeneration temperature (T°C) in	Input neurons	Output neuron	Hidden layer neuron	Goal	MSE	R
12	70	12	1	632	0.01	0.0144943	0.99739
14	50	12	1	632	0.01	0.0144943	0.99442
14	60	12	1	632	0.01	0.0144943	0.98924
14	70	12	1	632	0.01	0.0144943	0.99711
14	80	12	1	632	0.01	0.0144943	0.99542
16	70	12	1	632	0.01	0.0144943	0.99636
18	70	12	1	632	0.01	0.0144943	0.98876

**Fig. 8:**
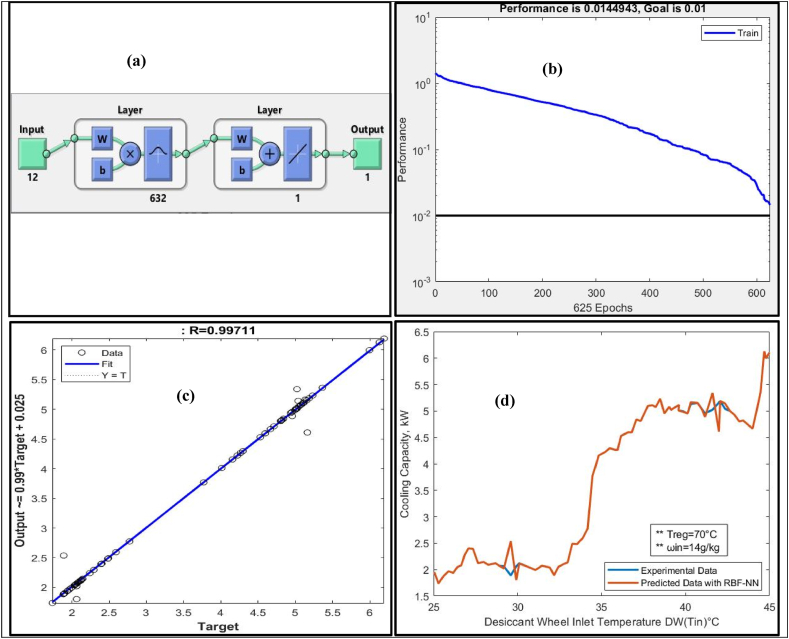
(a) Architecture of RBF-NN (b) Performance Curve (c) Regression plot (d) Comparison between Predicated and Experimental data.

RBF-NN showed the best prediction of CC for Sol-DAC measured as regression (R = 0.99711), when supply air humidity ratio was (14 g/kg) and the regeneration temperature for the Sol-DAC system was at 70 °C. Fig. 8(a) shows the architecture of the trained RBF-NN model that consists of 12 input neurons, 1 output neuron, and chooses 632 hidden layer neurons to achieve the preset Goal MSE of 0.01. Fig. 8(b) shows the convergence curve of the RBF-NN against the preset MSE. The blue line shows the trend of reduction in MSE during training of the RBF-NN as epochs pass. It can be observed from the figure that the network took 625 epochs to achieve the preset MSE of 0.01. Fig. 8(c) shows the regression plot between predicted CC for the Sol-DAC using RBF-NN after testing and their corresponding targets from the experimental data. It is evident from the regression plot (R = 0.99711) that most of the predicted data points lies very close to the fitting line except few outliers.

After predicting the CC of the Sol-DAC system using trained RBF-NN, experimental and predicted data were simultaneously plotted to check the performance of the network as a regression model in Fig. 8(d). The predicted CC of the Sol-DAC using RBF-NN is in close agreement with the CC of the Sol-DAC calculated by experimental data and follows the same trend. Fig. 8(d) shows that the Sol-DAC system tested in the temperature range of 25–45 °C and humidity ratio of 12–18 g/kg at the inlet of the desiccant wheel of Sol-DAC. It is clear from Fig. 8(d) that when the supply air temperature rises, the CC of the Sol-DAC system is raised. The evaporative cooler efficiency of the system is significantly increased at higher input temperatures, even while dehumidification efficiency falls as inlet humidity increases. CC represents the sensible load handling capacity of the system and mainly depends on the inlet supply temperature and regeneration temperature as well. Regeneration temperature increases the effectiveness of DW for dehumidification lower the humid air is easy to handle for cooling purpose.

## 7 Conclusions

Integration of solar assisted solid desiccant system with M-cycle indirect evaporative cooler is proposed to achieve the thermal comfort level. Silica-gel was used as desiccant material for rotary desiccant wheel. Extensive experimental data was collected in different months by changing the input ranges of different parameters; it was found that the Sol-DAC system is capable of providing outlet conditions to achieve the thermal comfort level. The desiccant wheel was used to control the latent load, and M-cycle was used to control the sensible load. Sol-DAC system was tested using inlet humidity range of 12–18 g/kg and inlet temperature range of 25–45 °C. Based on the experimental analysis it is concluded that Sol-DAC system integrated with M-cycle is proven as more temperature effective as compared with DEC systems. Moreover, due to the exclusivity of the M-Cycle indirect evaporative cooler, there is no addition of moisture in the supply air. It is concluded from the results that the system operates well even in hot and humid climates due to the effectiveness of the M-cycle. Moreover, it is observed that due to the use of M-cycle, the whole Sol-DAC system is around 60–65 % more efficient than the other desiccant air conditioning system in terms of COPth providing the same inlet conditions and regeneration temperature.

Machine learning based data driven model has been established to predict the performance parameters of the designed Sol-DAC system. RBF-NN was trained as a regression tool to predict the four outlet parameters (T_out_, ω_out_, CC, and COP) of the Sol-DAC system that conforms to the thermal comfort levels. Experimental and predicted performance parameters were in close agreement and showed minimal deviation. Investigations of predicted results revealed that the trained RBF-NN model was capable of predicting the trend of output results under varying input conditions.

By comparison study of experimental and predicated results, it was found that the best MSE obtained for Tout, ωout, COP, and CC were 0.00998279, 0.0102932, 0.0106691, 0.0144943, respectively. These results of MSE showed that the accuracy of the RBF-NN model was satisfactory and close to the experimental values.

## 8 Discussion and future work

Due to the combination of DW and M-cycle and the assistance of a solar thermal system, Sol-DAC is an attractive package for air conditioning purposes. It has great potential to replace traditional air conditioning systems. Sol-DAC system has the potential to provide thermal comfort conditions in hot and humid climates but improvement in the geometry and material of M-cycle can enhance the system performance. Using an M-Cycle-based DAC system can save the emission of CO_2_ by about 24 %. Sol-DAC system has a higher capital cost than vapor compression system, but the running cost is very low as no compressor is needed in this system. Due to low electric consumption and the provision of human thermal conditions, the Sol-DAC system acceptance rate is high. One of the major drawbacks of this system is its large size because of the desiccant wheel and solar thermal system. The most effective way to optimize the working of an evaporative cooling based on the M-cycle is to adjust the ratio of the mass flow rate of air in the dry and wet channels. Leakages of air should be avoided, and the distribution of water and air systems should be properly designed to get better results.

Sol-DAC systems can be used in residentials sector as well in commercial buildings like offices, labs, cinemas etc where air conditioning is needed to fulfill the demand of human comfort level. DW is used to control humidity, many industries like pharmaceutical, surgical, textile and food industry needed to remove the humidity in ware houses so this system is excellent option for such types of industries. Moreover, besides the HVAC systems, M-Cycle can also be used in power generation plants for improving efficiency of the power plant with reduction NOx. Effective water desalination is another application of the M-cycle.

Applying the machine learning technique in real time Sol-DAC system can predict the desired responses of the system using training data, this may help the manufacturers to employ the ANN technique to evaluate the performance of Sol-DAC system, which can be economical also saves efforts of engineers.

## Data availability statement

Data will be made available on request.

## CRediT authorship contribution statement

**Sibghat Ullah:** Writing – original draft, Visualization, Validation, Methodology, Investigation, Formal analysis, Data curation, Conceptualization. **Muzaffar Ali:** Supervision, Resources, Project administration, Methodology, Conceptualization. **Muhammad Fahad Sheikh:** Writing – review & editing, Software, Investigation, Data curation. **Ghulam Qadar Chaudhary:** Writing – review & editing, Resources, Formal analysis, Data curation. **Laoucine Kerbache:** Writing – review & editing, Visualization, Supervision, Project administration, Funding acquisition.

## Declaration of competing interest

The authors declare that they have no known competing financial interests or personal relationships that could have appeared to influence the work reported in this paper.
